# Facile Electrochemical Sensor for Nanomolar Rutin Detection Based on Magnetite Nanoparticles and Reduced Graphene Oxide Decorated Electrode

**DOI:** 10.3390/nano9010115

**Published:** 2019-01-18

**Authors:** Quanguo He, Yiyong Wu, Yaling Tian, Guangli Li, Jun Liu, Peihong Deng, Dongchu Chen

**Affiliations:** 1 School of Life Sciences and Chemistry, Hunan University of Technology, Zhuzhou 412007, China; hequanguo@126.com (Q.H.); wyy5082010@163.com (Y.W.); tianyaling0212@163.com (Y.T.); guangli010@hut.edu.cn (G.L.); liu.jun.1015@163.com (J.L.); 2 Hunan Provincial Key Laboratory of Functional Metal-Organic Compounds; Key Laboratory of functional Organometallic Materials of Hunan Provincial Universities; Department of Chemistry and Material Science, Hengyang Normal University, Hengyang 421008, China; 3 School of Materials Science and Energy Engineering, Foshan University, Foshan 528000, China

**Keywords:** rutin, amine-functionalized Fe_3_O_4_ nanoparticles, electrochemically reduced graphene oxide, nanomolar electroanalysis

## Abstract

A new electrochemical sensor for nanomolar rutin detection based on amine-functionalized Fe_3_O_4_ nanoparticles and electrochemically reduced graphene oxide nanocomposite modified glassy carbon electrode (NH_2_-Fe_3_O_4_ NPs-ErGO/GCE) was fabricated through a simple method, and the X-ray diffraction (XRD), Fourier transform infrared spectroscopy (FTIR), scanning electron microscope (SEM), transmission electron microscope (TEM), vibrating sample magnetometer (VSM) and electrochemical technique were used to characterize the modified electrode. The electrochemical behavior of rutin on the Fe_3_O_4_ NPs-ErGO/GCE was studied in detail. The electrochemical response of rutin at this modified electrode was remarkably higher than that of the bare GCE or other modified GCE (GO/GCE, Fe_3_O_4_ NPs-GO/GCE, and ErGO/GCE). Under the optimum determination conditions, Fe_3_O_4_ NPs-ErGO/GCE provided rutin with a broader detection range of 6.0 nM–0.1 µM; 0.1–8.0 µM and 8.0–80 µM, a minimum detectable concentration of 4.0 nM was obtained after 210 s accumulation. This novel method was applied in determination of rutin in pharmaceutical tablets and urine samples with satisfactory results.

## 1. Introduction

Flavonoids are a kind of natural product widely existing in plants. They have antioxidant and anti-free radical effects and can be used in the treatment of cardiovascular and cerebrovascular diseases, tumors, inflammation and so on. Rutin (3’,4’,5,7-tetrahydroxyflavone-3-d-rutinoside), also known as vitamin P, is a common flavonoid glycoside widely present in many plants, especially in buckwheat plants [1]. Modern pharmacological studies have found that rutin not only regulates the permeability and fragility of the capillary wall, prevents vascular rupture and hemostasis, but also has the effects of anti-bacterial, anti-inflammatory, analgesic, anti-radiation, anti-oxidation, anti-myocardial hypoxia, reducing serum cholesterol, diuresis, spasmolysis and inhibiting platelet aggregation [2,3]. Therefore, it is of great significance to develop simple, reliable and sensitive methods for rutin analysis.

So far, numerous analysis methods, such as high performance liquid chromatography [4,5], chemiluminescence [6,7], capillary electrophoresis [8], spectrophotometry [9], flow injection analysis [10] and electrochemistry [11] have been reported for the determination of rutin. Among these methods, some involve expensive equipment, a time-consuming process, the use of toxic organic reagents or poor sensitivity. Electrochemical methods have the virtues of low-cost apparatus, high sensitivity, wide linear range and simplicity. Due to the electroactivity of rutin, various electrodes have been modified and explored for the detection of rutin [12,13,14,15,16,17,18,19,20], and their typical analytical figures of merit for rutin analysis are compared and summarized in Table 1. In order to amplify the response signal fundamentally and improve the detection sensitivity practically, it is still important and necessary to develop new electrochemical sensors for rutin detection at the nanomolar level.

Since graphene (GR) was first reported in 2004, this two-dimensional carbon nanomaterial has attracted tremendous attention due to its excellent properties such as high surface area, fast electron transportation, excellent mechanical strength, high thermal conductivity and high mobility of charge carriers [21]. In recent years, great progress has been made in GR based nanocomposites by utilizing the synergistic effect of the components used. GR has been combined with different material entities such as metal, metal oxide, biomolecules and polymers, and the nanocomposites can provide their individual advantages of each component and demonstrate an improved performance [22,23,24,25,26,27,28,29,30]. Fe_3_O_4_ nanoparticles (Fe_3_O_4_ NPs) are widely used in the biomedical field because of their good biocompatibility, low cost, easy preparation, large surface area and distinct catalytic activity [31,32,33]. However, Fe_3_O_4_ NPs exhibit poor performance on electrical conductivity and are inclined to agglomerate, which hampers their application in electrochemical sensors [34]. The fabrication and application of GR and Fe_3_O_4_ NPs composite has been explored recently. For example, Zhang et al. fabricated a Nafion covered core-shell structured Fe_3_O_4_@graphene nanospheres modified glassy carbon electrode (GCE) and used it for selective detection of dopamine [35]. Yin et al. prepared a nanosized graphene, chitosan and Fe_3_O_4_ composite as electrode material which exhibited an excellent electrocatalytic and adsorptive activity towards the oxidation of guanosine [36]. Teymourian et al. synthesized GR/Fe_3_O_4_ nanohybrids via a facile one-step chemical reaction strategy, where the reduction of graphene oxide (GO) and the in-situ generation of Fe_3_O_4_ nanoparticles occurred simultaneously, which showed good electrochemical behaviors toward the oxidation of NADH [37]. Sun et al. prepared various shapes (sphere, rod and band) Fe_3_O_4_ decorated reduced graphene oxide (rGO) via one-step co-precipitation route in solution. It was proved that the shapes of Fe_3_O_4_ had a great effect on sensing performance. The reason may be that their morphology had an important impact on the surface area, active site and ion kinetics of materials, which could effectively influence its electrochemical performance. The experimental data showed that the band-shaped Fe_3_O_4_/rGO modified electrode exhibited high sensitivity for Pb(II) detection [38]. However, up to now, the preparation of electrochemical sensors which combine the outstanding merits of Fe_3_O_4_ NPs and GR for rutin determination is still scarcely reported.

In this paper, an inexpensive, rapid and efficient synthetic route is adopted for the preparation of the Fe_3_O_4_ NPs-GR nanocomposite. Amine-functionalized Fe_3_O_4_ (NH_2_-Fe_3_O_4_ NPs) was synthesized, which could bind with GO via hydrogen bonding between the amino-groups attached to NH_2_-Fe_3_O_4_ and the oxygen-containing groups such as hydroxyl, epoxy and carboxyl groups residing on the surface of GO, and the resulting nanocomposites had good dispensability and stability. Then, the potentiostatic reduction of GO sheets was electrochemically employed to generate reduced graphene oxide (ErGO) on the surface of GCE. Based on this, cyclic voltammetry (CV) and second derivative linear sweep voltammetry (SDLSV) were both adopted to investigate as-fabricated NH_2_-Fe_3_O_4_ NPs-ErGO/GCE and its sensing performances towards the rutin oxidation. The electrochemical results showed that the voltammetric response of rutin was enhanced greatly on NH_2_-Fe_3_O_4_ NPs-ErGO/GCE. Therefore, a novel sensitive voltammetric approach for rutin determination at nanomolar level was symmetrically explored.

## 2. Experimental

### 2.1. Materials and Reagents

Rutin (C_27_H_20_O_16_·3H_2_O), sodium nitrate, concentrated sulfuric acid, ferric trichloride hexahydrate (FeCl_3_·6H_2_O), potassium permanganate, hydrogen peroxide, graphite powder, 1,2-ethylenediamine and ethylene glycol were purchased from Sinopharm Chemical Reagent Co. Ltd. (Shanghai, China). A stock solution of rutin (1.0 mM) was prepared in 50% (*v*/*v*) ethanol and kept at 4 °C when not in use. More dilute solutions were prepared accordingly when needed. For all electrochemical measurements, 0.2 M H_2_SO_4_ was used as the electrolyte. All other reagents were of analytical grade and used as received. Doubly distilled water was used throughout the study.

### 2.2. Characterization Apparatus

Cyclic Voltammetry (CV) was measured on a CHI 660E electrochemical workstation (Chenhua Instrument, Shanghai, China). Second derivative linear sweep voltammetry (SDLSV) was conducted on a JP-303E polarographic analyzer (Chengdu Instrument Factory, Chengdu, China). A conventional three-electrode system was used throughout the electrochemical experiments, which consisted of a working electrode (NH_2_-Fe_3_O_4_ NPs-ErGO/GCE), a counter electrode (platinum electrode), and a reference electrode (saturated calomel electrode, SCE), respectively. The pH measurements were performed on a pH-3c exact digital pH meter (Shanghai Leichi Instrument Factory, Shanghai, China). Fourier transform infrared spectroscopy (FTIR) was recorded on Varian Excalibur 3100 spectrometer (Palo Alto, CA, USA). Scanning electron microscope (SEM) images were collected from a Hitachi S-3000N scanning electron microscope (Hitachi, Tokyo, Japan) at an acceleration potential of 30 kV. Transmission electron microscope (TEM) images were recorded from JEOL JEM-2010 (HT, Tokyo, Japan) operated at 200 kV. X-ray diffraction (XRD) patterns were operated with an X-ray diffractometer (PAN Alytical, Amsterdam, Holland). Magnetic hysteresis loop of NH_2_-Fe_3_O_4_ NPs was detected by a vibrating sample magnetometer (VSM, Instrument Factory of Nanjing University, Nanjing, China). The ultraviolet (UV) absorption spectra were obtained on a UV-2501PC spectrophotometer (Shimadzu Co., Tokyo, Japan) in the wavelength range 200–450 nm with 1 cm matched quartz cells.

### 2.3. Preparation of NH_2_-Fe_3_O_4_ NPs and GO Composites

Graphene oxide (GO) was synthesized using a modified Hummers method as reported in our previous publication [39]. Briefly, 23 mL concentrated sulfuric acid was cooled to 0 °C, and 0.5 g graphite powder, 0.5 g sodium nitrate and 3.0 g potassium permanganate were added under mechanical stirring below 5 °C. Then, the mixture was stirred for 2 h at 35 °C to form a mash. Subsequently, 40 mL water was added slowly into the solution below 50 °C, then the temperature was increased to 95 °C and the mixture was stirred for 0.5 h. After adding 100 mL of water, the above solution was poured in 20 mL of 30% H_2_O_2_. The suspension was treated by suction filtration, and the precipitate was washed with 1.0 M hydrochloric acid and water, respectively. The graphite oxide was obtained by drying under 50 °C vacuum overnight. Finally, 100 mg graphite oxide were dispersed in 100 mL water and exfoliated to GO by ultrasonication for 2 h, and the golden-yellow solution was obtained after centrifugation. 

NH_2_-Fe_3_O_4_ NPs were prepared via hydrothermal method according to [40]. In brief, 1.0 g FeCl3·6H2O was dissolved in 20 mL ethylene glycol to form a clear solution, followed by the addition of 3.0 g sodium acetate, 0.4 g sodium hydroxide, and 10 mL 1,2-ethylenediamine. The mixture was stirred vigorously for 30 min and then sealed in a Teflon lined stainless-steel autoclave. The autoclave was heated to and maintained at 200 °C for 8 h, and allowed to cool to room temperature. The obtained black magnetite particles were washed with water for several times and then dried in vacuum. After that, 1.0 mg of NH_2-_Fe_3_O_4_ NPs was introduced into 20 mL of GO solution (1 mg/mL) in an ultrasound bath for 2 h and the NH_2_-Fe_3_O_4_ NPs and GO nanocomposites dispersed solution were produced.

### 2.4. Fabrication of the Decorated Electrodes

In order to acquire a smooth surface, the bare GCE was polished with 0.05 µm α-Al_2_O_3_ suspension, and then it was cleaned in ethyl alcohol and doubly distilled water by sonication, each for 2 min. A total of 5 µL of NH_2_-Fe_3_O_4_ NPs and GO nanocomposites dispersion was dropwise added onto the GCE surface and dried under an infrared lamp. The electrode denoted as NH_2_-Fe_3_O_4_ NPs-GO/GCE was achieved. Then, NH_2_-Fe_3_O_4_ NPs-ErGO/GCE was fabricated by electrochemical reduction of GO in phosphate buffer solution (PBS, pH 6.5) at −1.2 V for 150 s. For comparison and further investigation, NH_2_-Fe_3_O_4_ NPs/GCE, GO/GCE, ErGO/GCE were similarly fabricated as above-mentioned procedure.

### 2.5. Analytical Procedure

A total of 1.0 mL of 0.1 mM rutin, 1.0 mL of 1.0 M hydrochloric acid and 8.0 mL of water were transferred into a 10 mL electrochemical cell, and then the three-electrode system was set up. The electrochemical behavior of rutin on NH_2_-Fe_3_O_4_ NPs-ErGO/GCE was measured by CV method, and SDLSV was used for rutin quantitative analysis because of its sensitivity and high resolution. CVs potential data were collected and recorded in the range from 0.0 to 1.0 V at a scan rate of 0.1 V/s. While SDLSVs potential data were collected and recorded from −0.3 to 1.1 V at a scan rate of 0.1 V/s similarly. All measurements were carried out at room temperature.

## 3. Results and Discussion

### 3.1. Characterization of Morphology and Structure of the Synthetic Materials

**Figure 1:**
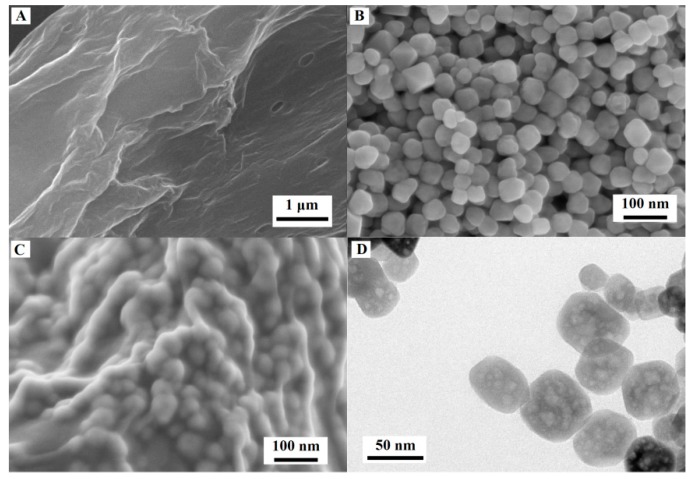
Scanning electron microscope (SEM) images of graphene oxide (GO) (**A**), Amine-functionalized Fe_3_O_4_ nanoparticles (NH_2_-Fe_3_O_4_ NPs) (**B**) and NH_2_-Fe_3_O_4_-electrochemically reduced graphene oxide (ErGO) nanocomposites (**C**) and the Transmission electron microscope (TEM) image of NH_2_-Fe_3_O_4_ NPs (**D**).

The morphologies of the synthesized materials were primarily characterized by SEM, and the images of GO (A), NH_2_-Fe_3_O_4_ NPs (B) and NH_2_-Fe_3_O_4_-ErGO nanocomposites (C) were obtained. As shown in Figure 1A, GO exhibited thin, wrinkled nanosheets. The typical morphology of NH_2_-Fe_3_O_4_ NPs was clearly presented in Figure 1B, in which NH_2_-Fe_3_O_4_ NPs exhibit a narrow size-distribution with an estimated average particle diameter of about 50 nm. The SEM image of NH_2_-Fe_3_O_4_ NPs-ErGO in Figure 1C shows that ErGO sheets decorated with NH_2_-Fe_3_O_4_ are stacked randomly, indicating that NH_2_-Fe_3_O_4_ NP and ErGO are well combined. In addition, the surface morphology of NH_2_-Fe_3_O_4_ NPs was studied by TEM. Most interestingly, these NH_2_-Fe_3_O_4_ NPs with smooth surfaces demonstrate a mesoporous structure inside, and the porous size is around 10 nm. It is assumed that the assembling similar-surfactant structures formed via static attraction between sodium acetate (excess) and 1, 2-ethylenediamine ETH (excess) probably acted as the soft template for obtaining the novel mesoporous structure [41]. The NH_2_-Fe_3_O_4_ NPs shows good dispersity and no obvious agglomeration is observed, plus the significantly small size and abundant pores, the specific surface area of NH_2_-Fe_3_O_4_ NPs increases dramatically. It is well known that large specific surface areas can provide more active sites and can absorb more analytes. Moreover, these pores also allow the electrons to transit inside their interior pore channels, which would improve electrocatalytic activity.

**Figure 2:**
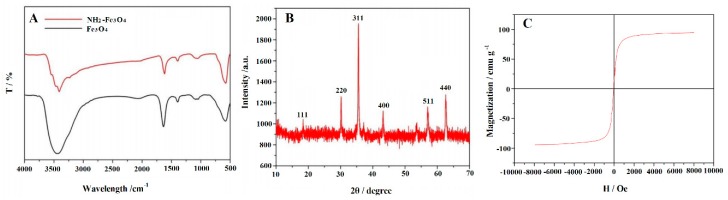
Fourier transform infrared spectroscopy (FTIR) spectra of Fe_3_O_4_ NPs and NH_2_-Fe_3_O_4_ NPs (**A**), the X-ray diffraction (XRD) pattern of NH_2_-Fe_3_O_4_ NPs (**B**) and the vibrating sample magnetometer (VSM) of NH_2_-Fe_3_O_4_ NPs (**C**).

NH_2_-Fe_3_O_4_ NPs were further characterized by FTIR, XRD and VSM separately. Figure 2A shows the FTIR spectra comparison of Fe_3_O_4_ NPs (curve a) and NH_2_-Fe_3_O_4_ NPs (curve b). From curve b, it could be seen that the broad band in the range of 3300–3600 cm^−1^ was attributed to stretching vibrations of hydroxyl (-OH groups) absorbed by Fe_3_O_4_ NPs, and the absorption band near 3211 cm^−1^ was attributed to the -NH groups also existing in particles. The strong absorption peak at 577 cm^−1^ was caused by the stretching vibrations of the Fe-O bond in NH_2_-Fe_3_O_4_ NPs. After amine-modification, the peak strength was weakened as compared with that of parent Fe_3_O_4_ NPs, which indicated that the modification of Fe_3_O_4_ NPs with -NH_2_ was successful. It was evident that the XRD patterns of NH_2_-Fe_3_O_4_ NPs (Figure 2B) displayed six diffraction peaks located at 2θ of 19.7°, 30.4°, 35.7°, 43.2°, 57.3° and 62.8°, respectively corresponding to the facets of (111), (220), (311), (400), (511) and (440) exactly the same as magnetite crystal structure (JSPDS01-1111, α = 8.393Å). No other impurity diffraction peaks were observed in the XRD pattern, indicating that the prepared NH_2_-Fe_3_O_4_ NPs were crystally pure. The magnetization curves of the NH_2_-Fe_3_O_4_ NPs are shown in Figure 2C, and their magnetic saturation Ms values are around 80 emu·g^−1^, quite similar to crystal magnetite NPs, demonstrating a very good soft magnetism [32].

### 3.2. Electrochemical Properties of Modified Electrodes

**Figure 3:**
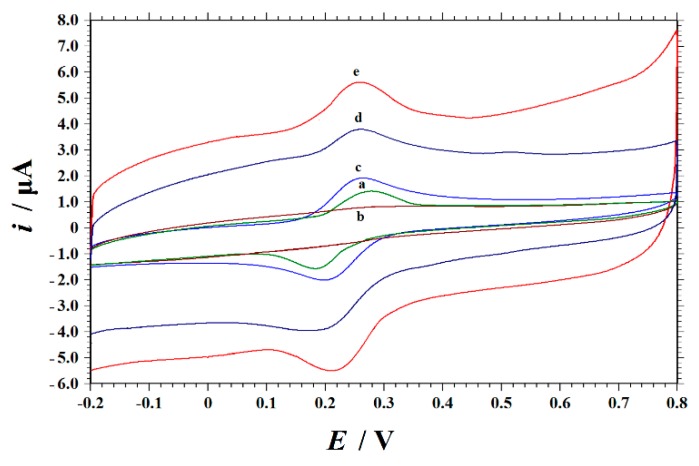
Cyclic voltammograms of glassy carbon electrode (GCE) (**a**), graphene oxide modified glassy carbon electrode (GO/GCE) (**b**), NH_2_-Fe_3_O_4_ NPs-GO/GCE (**c**), ErGO/GCE (**d**), and NH_2_-Fe_3_O_4_ NPs-ErGO/GCE (**e**) in 1.0 mmol/L K_3_[Fe(CN)_6_] and 0.5 mol/L KCl with the scan rate of 0.1 V/s.

The cyclic voltammetric responses on different electrodes were compared and recorded in a mixed solution of 1.0 mM K_3_[Fe(CN)_6_] and 0.5 M KCl concentrations, as shown in Figure 3. On GCE the peak-to-peak potential separation (Δ*E*_p_) was 79 mV and the redox peak current is the smallest (curve a), implying the quasi-reversible electron transfer process involved. However, after GO modification, there was a significant decrease of the redox peak current of [Fe(CN)_6_]^3−/4−^, which may be caused by the weak conductivity of GO and negative charge repulsion force between the ionized groups such as COO- in GO and [Fe(CN)_6_]^3−/4−^ (curve b). While on NH_2_-Fe_3_O_4_ NPs-GO/GCE (curve c) and ErGO/GCE (curve d) the redox peak currents increased gradually, which may result from the presence of NH_2_-Fe_3_O_4_ NPs and ErGO on GCE surface. Moreover, the current value on ErGO/GCE was larger than that of NH_2_-Fe_3_O_4_ NPs/GCE, indicating a better conductivity of ErGO nanosheets than that of NH_2_-Fe_3_O_4_ NPs. On NH_2_-Fe_3_O_4_ NPs-ErGO/GCE (curve e), the highest redox peak current appeared with the Δ*E*_p_ value decreased to 42 mV, implying a more reversible electron transfer process occurred for [Fe(CN)_6_]^3−/4−^ probe. Therefore, the coexistence of ErGO and NH_2_-Fe_3_O_4_ NPs on the surface of GCE can complementarily improve the electron transfer rate and optimize a suitable electrochemical interface for the further application.

### 3.3. Rutin Cyclic Voltammograms on the Decorated Electrodes

**Figure 4:**
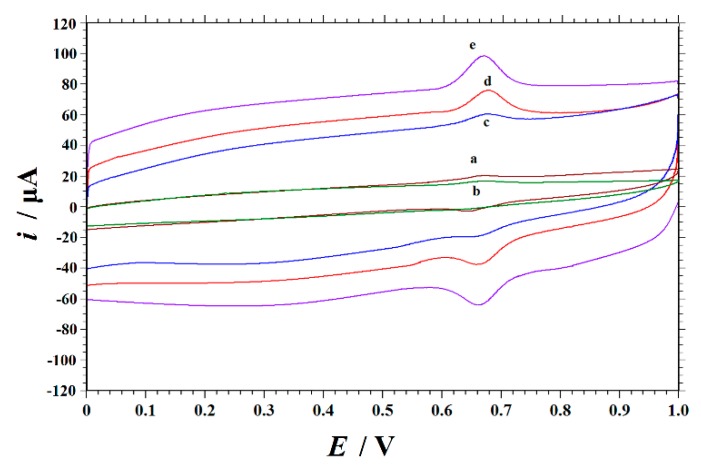
Cyclic voltammograms of 10.0 µM rutin on the different electrodes in 0.2 M H_2_SO_4_: GCE (**a**), GO/GCE (**b**), NH_2_-Fe_3_O_4_ NPs-GO/GCE (**c**), ErGO/GCE (**d**), and NH_2_-Fe_3_O_4_ NPs-ErGO/GCE (**e**). The scan rate of 0.1 V/s.

The electrochemical behavior of rutin was firstly investigated by means of cyclic voltammetry (CV). Figure 4 shows the cyclic voltammograms of 10.0 µM rutin at different electrodes in 0.2 M H_2_SO_4_ at a scan rate of 0.1 V s^−1^. A pair of redox peaks was observed at each electrode from 0.0 to 1.0 V, indicating that rutin underwent a reversible redox process. Table 1 summarized the rutin electrochemical data at various decorated electrodes. It can be seen that on GO/GCE, the redox peak currents were smaller than that of GCE (curve b), which may be due to the low conductivity of GO, resulting in delayed electron transfer. The electrochemical data showed that the redox currents of rutin on NH_2_-Fe_3_O_4_ NPs-GO/GCE (curve c) and ErGO/GCE (curve d) were larger than that of bare GCE (curve a), which indicated the superiority of NH_2_-Fe_3_O_4_ NPs-GO/GCE and ErGO/GCE such as large surface area, inherent electrocatalytic ability, and good conductivity towards rutin detection. While on NH_2_-Fe_3_O_4_ NPs-ErGO/GCE the maximum redox peak currents appeared, indicating the synergistic effects of ErGO and NH_2_-Fe_3_O_4_ NPs were present on the electrode surface. ErGO has the advantages of large specific surface area, good catalytic activity and high conductivity. While the NH_2_-Fe_3_O_4_ NPs used in this experiment have excellent electrocatalytic activity and can be used as an electronic mediator to promote the transfer of electrons between the modified electrode and rutin solution. Due to the excellent properties of ErGO and NH_2_-Fe_3_O_4_ NPs combination, the sensing performance of the sensor towards rutin detection was greatly improved.

**Figure 5:**
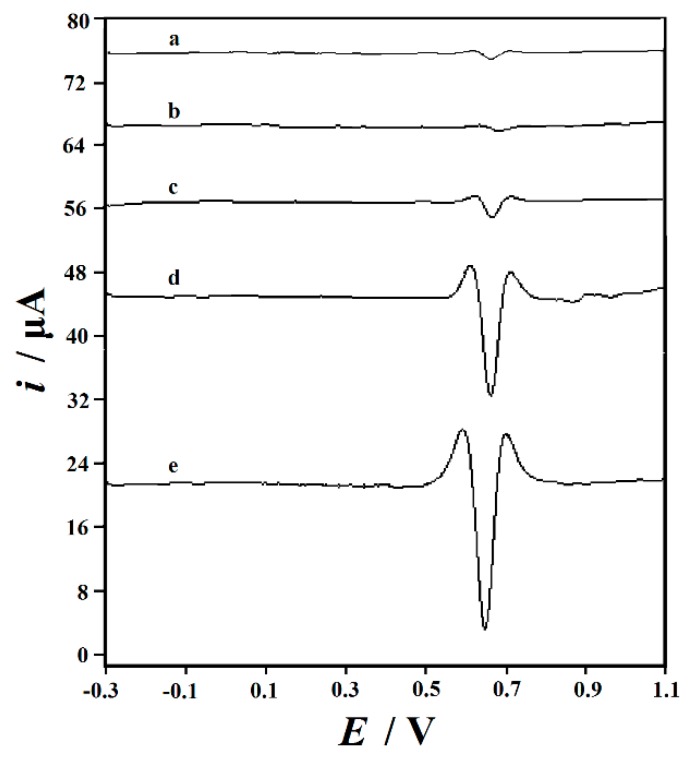
Second-order derivative linear scan voltammograms of 10.0 µM rutin on the different electrodes in 0.2 M H_2_SO_4_ after accumulation at −0.3 V for 210 s: GCE (**a**), GO/GCE (**b**), NH_2_-Fe_3_O_4_ NPs-GO/GCE (**c**), ErGO/GCE (**d**), and NH_2_-Fe_3_O_4_ NPs-ErGO/GCE (**e**). The scan rate of 0.1 V/s.

The electrochemical behavior of rutin on various electrodes was also investigated by the method of second derivative linear sweep voltammetry (SDLSV) in order to improve the sensitivity. Figure 5 shows the corresponding voltammograms of the NH_2_-Fe_3_O_4_ NPs-ErGO/GCE immersed in 10.0 µM rutin (0.2 M H_2_SO_4_). As it can be noticed, the oxidation peaks appear to have the same potentials as in the case of the CV. As compared to those of the CV results, the peaks obtained via SDLSV are better defined, the background current is lower, and the currents are higher. Therefore, SDLSV is the most suitable technique for rutin quantitative analysis. As shown in Figure 5, the current responses of rutin on different electrodes increased in the order of *i*_p_ (GO/GCE) (0.6250 μA) < *i*_p_ (GCE) (1.863 μA) < *i*_p_ (NH_2_-Fe_3_O_4_ NPs-GO/GCE) (2.650 μA) < *i*_p_ (ErGO/GCE) (15.99 μA) < *i*_p_ (NH_2_-Fe_3_O_4_ NPs-ErGO/GCE) (24.95 μA). As compared with that of GCE, the oxidation peak potential of rutin on NH_2_-Fe_3_O_4_ NPs-ErGO/GCE shifted negatively by 11 mV and the oxidation peak current increased by nearly 13 times, as a result of the synergistic enhancement effect of ErGO and NH_2_-Fe_3_O_4_ NPs on the electrochemical reaction of rutin.

### 3.4. Effect of Scan Rate

**Figure 6:**
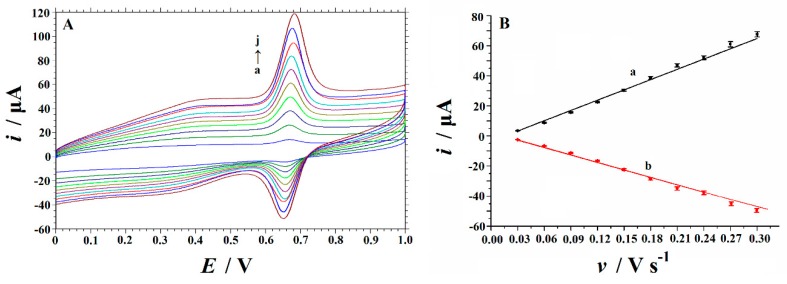
(**A**) Cyclic voltammograms of 10 µM rutin in 0.1 mol L^−1^ HCl solution on NH_2_-Fe_3_O_4_ NPs-ErGO/GCE at various scan rates (Form a to j: 0.03, 0.06, 0.09, 0.12, 0.15, 0.18, 0.21, 0.24, 0.27, 0.3 V s^−1^, respectively); (**B**) Plot of oxidation peak currents (**a**) and reduction peak currents (**b**) versus scan rates.

Figure 6 shows the cyclic voltammograms of 10.0 µM rutin at NH_2_-Fe_3_O_4_ NPs-ErGO/GCEABPE with different scan rates (0.03–0.3V/s). As exhibited in Figure 6A, a pair of roughly symmetric anodic and cathodic peaks appeared, the rutin peak currents increased as the scan rate (*v*) increased and a significant linear relationship is observed between the peak currents and scan rates (Figure 6B). The regression equation is *i*_pa_ (µA) = 253.01 *v* (V/s) −6.4469 (R^2^ = 0.9958) and *i*_pc_ (µA) = −187.86*v* (V/s) + 4.9146 (R^2^ = 0.9931), indicating that the electrode process of rutin on NH_2_-Fe_3_O_4_ NPs-ErGO/GCE is controlled by adsorption. However, there was no significant change in the peak potentials with increasing the scan rate, which confirmed that the electrochemical reaction of rutin is reversible on the surface of NH_2_-Fe_3_O_4_ NPs-ErGO/GCE.

### 3.5. Effects of Supporting Electrolyte and Solution pH Variation

In general, supporting electrolyte and the pH of buffer solution play a dominant role for the voltammetric response of electrochemically active substances. To understand the effect of supporting electrolyte, SDLSVs were recorded and compared in many buffer solutions including HAc–NaAc buffer (pH 4.0), HAc-NH_4_Ac buffer (pH 4.0), phosphate buffer (pH 6.0), (CH_2_)_6_N_4_-HCl buffer (pH 5.0), NH_3_-NH_4_Cl (pH 9.0), H_2_SO_4_, HNO_3_, HCl and NaOH, each 0.1 M), respectively. The experimental data in Table 2 proved that the electro-oxidation of rutin is better performed in acidic media, while in neutral or alkaline media, the peak current decreased considerably. These phenomena might be explained by the fact that the hydroxyl groups in the rutin structure lost their protons in the basic solution, so the electrochemical reaction of rutin was more difficult to achieve [14,42]. The highest peak current and the best peak shape were observed in H_2_SO_4_ solution, thus it was selected as the working media. 

**Scheme 1:**
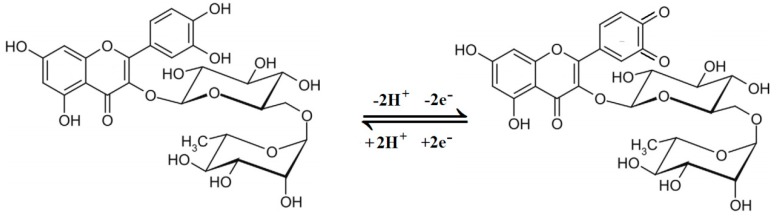
Electrochemical reaction mechanism of rutin.

**Figure 7:**
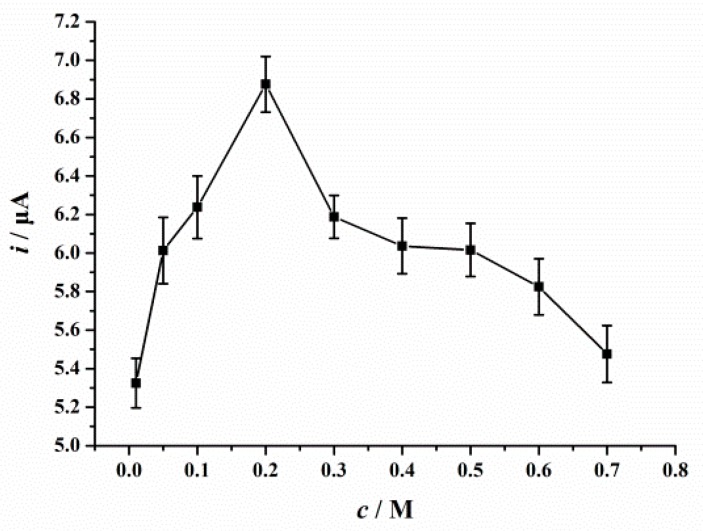
The dependence relationship of the peak current of 10 µM rutin and H_2_SO_4_ concentration by SDLSV, error bars represent SD, *n* = 3. Accumulation potential: −0.3 V, accumulation time: 30 s, scan rate of 0.1 V/s.

From Figure 7, it can be seen that the oxidation peak current of rutin increased with the increase of H_2_SO_4_ concentration from 0.01 to 0.2 M, and then decreased with further increasing the concentration from 0.2 to 0.7 M. The maximum peak current appeared at 0.2 M concentration of H_2_SO_4_. The influence of pH on the peak potential of rutin was also studied. By changing the concentrations of H_2_SO_4_, different pH values media were obtained. As the value of the pH rises, a linear movement towards the lower peak potential values can be observed, and corresponding regression equation is *E*_pa_ (V) = −0.0556 pH + 0.6528 (R^2^ = 0.9862). The slope between the peak potential and the pH is −55.6 mV/pH units, indicating that an equal number of electrons and protons are involved in the redox process as same as reported previously [43]. According to [13,14,42], the mechanism of electrooxidation of rutin could be explained as that the catechol structure of rutin is oxidized to 3’,4’ -diquinone, and the reduction reaction could take place on the decorated electrode surface. The electrode reaction equation was illustrated as the following Scheme 1.

### 3.6. Optimization of Parameters

#### 3.6.1. Electrochemical Reduction Conditions

**Figure 8:**
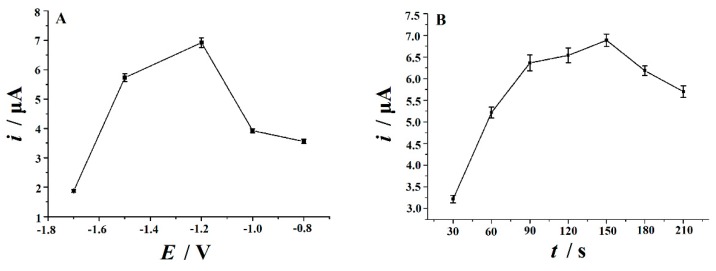
(**A**) Effect of reduction potential and (**B**) reduction time on the peak current of 10 µM rutin in 0.2 M H_2_SO_4_ at NH_2_-Fe_3_O_4_ NPs-ErGO/GCE by second derivative linear sweep voltammetry (SDLSV). Other conditions are the same as in Figure 7.

It is well known that pure GR sometimes fails to demonstrate perfect performance because oxygen-contaminated functional groups (OxFG) often play a leading role of catalytic active centers in electrochemical reactions as reported [44,45]. The potentiostatic reduction method is easy to control. By changing the potential and time of reduction, a certain number of OxFG can remain [44]. In this work, GO was reduced by potentiostatic method. The NH_2_-Fe_3_O_4_ NPs-ErGO/GCE was immersed and reduced in phosphate buffer (pH 6.5) under adjusting potentials (−0.8, −1.0, −1.2, −1.5, and −1.7 V, respectively) for 120 s. After that, the oxidation peak currents of 10 µM rutin were measured and compared. The results showed that the peak current of rutin increased with the decrease of reduction potential, from −0.8 to −1.2 V. However, the peak current decreased when the reduction potential shifted to more negative direction (Figure 8A). The effect of reduction time was also investigated at a constant reduction potential of −1.2 V (Figure 8B). The peak current increased greatly within the first 150 s and then declined gradually. Consequently, −1.2 V and 150 s were used for the electrochemical optimum conditions for GO reduction.

#### 3.6.2. Accumulation Conditions

**Figure 9:**
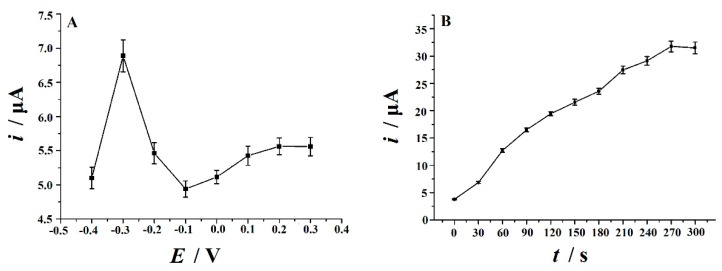
(**A**) Accumulation potential effects and (**B**) accumulation time effects on the peak current of 10 µM rutin in 0.2 M H_2_SO_4_ at NH_2_-Fe_3_O4 NPs-ErGO/GCE by SDLSV.

To enrich rutin concentration onto the NH_2_-Fe_3_O_4_ NPs-ErGO/GCE surface, accumulation was carried out before its detection. The effect of accumulation potential and time on the oxidation peak current (*i*_pa_) were examined. After 30 s accumulation time, the *i*_pa_ was recorded at varying accumulation potentials in the range from −0.4 to 0.3 V. As shown in Figure 9A, the highest *i*_pa_ appeared at the accumulation potential of −0.3 V. What is more, the effect of accumulation time was studied and compared at a fixed accumulation potential of −0.3 V (Figure 9B). In the first 270 s, with the increase of accumulation time, the *i*_pa_ increased significantly, and then with the further increase of accumulation time, the *i*_pa_ remained basically unchanged, indicating that the adsorption reached saturation. However, it is inconvenient using a long accumulation time. Therefore, the accumulation time of 210 s was used as a compromise between analysis speed and sensitivity.

### 3.7. Figures of Merit

#### 3.7.1. The Study of Interferences

In order to facilitate the application, the effects of some foreign substances, especially those that commonly co-exist in biological samples and the pharmaceutical formulations, on the rutin detection were investigated by SDLSV. The interference study was performed by immersing the NH_2_-Fe_3_O_4_ NPs-ErGO/GCE into a fixed concentration of 1.0 μM rutin solution mixed with added foreign species. The results demonstrated that the relative error (RE) after adding interfering substances was less than ±5.0%, which indicated that NH_2_-Fe_3_O_4_ NPs-ErGO/GCE has good anti-interference ability for rutin determination (Table 3).

**Figure 10:**
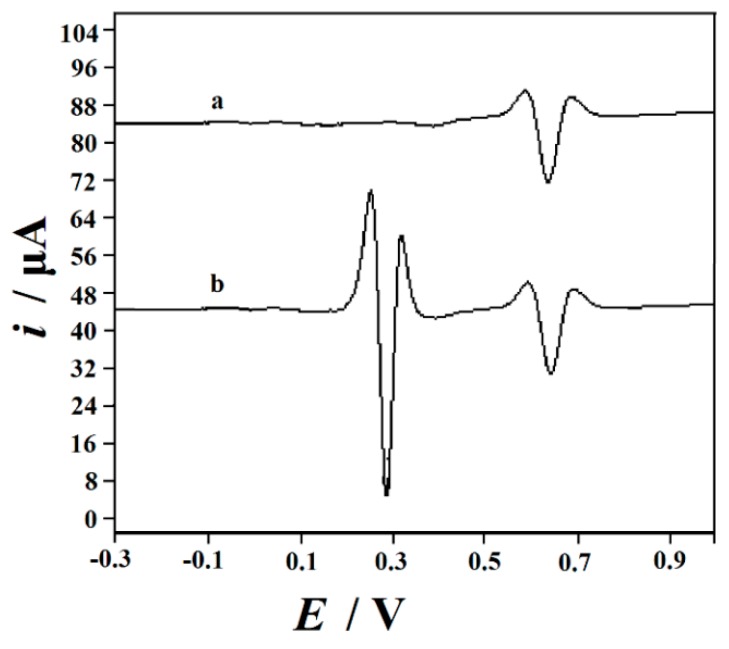
Second-order derivative linear scan voltammograms of 10 µM rutin in the absence of ascorbic acid (AA) (**a**) and in the presence of 1.0 mM AA (**b**) on NH_2_-Fe_3_O_4_ NPs-ErGO/GCE in 0.1 M HCl after a 120 s accumulation at −0.3V. Scan rate is 0.1 V s^−1^.

In addition, ascorbic acid (AA) is a chemically electroactive substance which often coexists with rutin in plants, crude drugs, pharmaceutical preparations (especially compound rutin tablets) and other biological samples. What is worse, the oxidation peak potential of AA is very close to that of rutin, indicating a poor selectivity and serious interfering effect for the rutin detection in real samples [19,20]. Therefore, the electrochemical responses of rutin in the presence of AA on the NH_2_-Fe_3_O_4_ NPs-ErGO/GCE were studied. The typical SDLSVs of 10.0 µM rutin in the absence and presence of AA on NH_2_-Fe_3_O_4_ NPs-ErGO/GCE is shown in Figure 10. A well defined oxidation peak at 0.665 V was found for rutin (curve a). Curve b is the voltammogram of 10.0 µM rutin in the presence of 1.0 mM AA under the same conditions. Two oxidation peaks of AA and rutin correspond to those at 0.296 and 0.665 V respectively. Thus, the oxidation peak potential separation was calculated as 0.369 V. The results show that a 100-fold excess of AA had no substantial interfering effect on the rutin determination (RE = −2.94%).

#### 3.7.2. Calibration and Detection Limit

**Figure 11:**
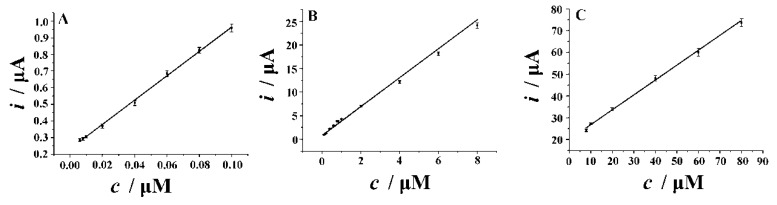
Plot of the peak current versus the concentration of rutin in the range from 6.0nM–0.1 µM (**A**); 0.1–8.0 µM (**B**) and 8.0–80 µM (**C**). Accumulation potential: −0.3 V, accumulation time: 210 s, scan rate of 0.1 V/s.

Under the optimized conditions and parameters, the calibration curve of rutin detection was established by SDLSV technique due to its better sensitivity and well-separated peak shape. The *i*_pa_ value of rutin increased gradually with the increment of rutin concentration. As plotted in Figure 11, the linear relationships were established in three sections from the range of 6.0 nM–0.1 µM; 0.1–8.0 µM and 8.0–80 µM, the linear regression equations were *i*_p_ (µA) = 7.3324*c* (µM) + 0.2308 (R^2^= 0.9986), *i*_p_ (µA) = 2.8677*c* (µM) + 1.0583 (R^2^= 0.9984), and *i*_p_ (µA) = 0.6752*c* (µM) + 20.021 (R^2^= 0.9982), respectively. A low detection limit of 4.0 nM was achieved. It is found that the calibration dependences exhibit intersection of calibration lines and the slopes are decreasing with increasing concentration of rutin. This phenomenon is caused by the fact that the peak current of rutin increases rapidly at low concentrations, and then increases slowly at higher concentrations. This may be explained with the saturated adsorption of rutin on the electrode surface and the rising of diffusion current at higher concentrations.

The method reported hereby and some previous procedures for rutin analysis are compared and summarized in Table 4 in terms of analytical figures and merits. As listed in Table 4, the proposed method can provide a linear range and detection limit comparable to (even superior to) other methods. In addition, this novel method has made remarkable progress in electrode fabrication simplification, time-saving and cost-reduction.

#### 3.7.3. Repeatability and Stability of the Decorated Electrode

The repeatability of the NH_2_-Fe_3_O_4_ NPs-ErGO/GCE was evaluated by detecting 10 μM rutin. The relative standard deviation (RSD) of peak current measured for seven consecutive times was 2.7%. Six different electrodes were fabricated under the same procedure and the reproducibility was also tested. The RSD was 6.2% for the peak current of 10 μM rutin. The stability of the modified electrode was also examined. The electrode was stored at 4 °C in a refrigerator when not in use, and the current response of 10 μM rutin on the electrode remained 92.1% (RSD = 2.7%, *n* = 3) of its initial value after a week. These results indicated that the modified electrode had good regeneration performance and stability.

### 3.8. Analytical Application

Compound rutin tablets (20 mg per tablet, Shanxi Yunpeng Pharmaceutical Co. Ltd., China; Shanxi Linfen Qilin Pharmaceutical Co. Ltd., Linfen, China) and urine samples from volunteers were detected in order to evaluate the reliability in practical application of the developed method. Five pieces of rutin tablets were ground into powder in an agate mortar, and then the accurate weight of one tablet was weighed, dissolved with absolute alcohol and diluted to 10.0 mL to obtain the stock solution. Subsequently, an appropriate amount of the stock solutions and urine samples were diluted with 0.1 M HCl by different factors as the testing solution. Electrochemical test was performed under the optimal experimental conditions by SDLSV and the recoveries were obtained by the standard addition method. As summarized in Table 5, the results were satisfactory with the recoveries in the range of 98.8–103.7% for tablet samples and 101.4–103.0% for urine samples, respectively. UV-vis spectrophotometry was selected as a reference method to assess the accuracy of the proposed method. The *t*-test implied that there is no significant difference when the results are at a 99% confidence level, which proved that the voltammetric method for rutin detection is precisely accurate for real sample analysis.

## 4. Conclusions

Herein, a feasible and reliable electrochemical sensor for the detection of rutin was developed using a nanocomposite of amine-functionalized Fe_3_O_4_ nanoparticles and electrochemically reduced graphene (NH_2_-Fe_3_O_4_ NPs-ErGO) as the sensing material. Compared with bare GCE, ErGO/GCE and NH_2_-Fe_3_O_4_ NPs-GO/GCE, the oxidation signals of rutin are significantly enhanced on the nanocomposites modified GCE (NH_2_-Fe_3_O_4_ NPs-ErGO/GCE). Moreover, this modified electrode has been employed successfully to eliminate the interferences of ascorbic acid. The linear range for the detection of rutin using NH_2_-Fe_3_O_4_ NPs-ErGO/GCE were 6.0 nM–0.1 µM; 0.1–8.0 µM and 8.0–80 µM. The detection limit was 4.0 nM (S/N = 3). The developed method is sensitive and selective for the determination of rutin in pharmaceutical tablets and urine samples. Moreover, this novel method has some obvious advantages such as quick response, simplicity, high sensitivity and low cost.

## Figures and Tables

**Table 1:** Electrochemical response data of rutin (10 µM) at different decorated electrodes.

Working Electrodes	i_pa_/µA	i_pc_/µA	E_pa_/V	E_pc_/V	i_pa_/i_pc_	ΔE_p_
^a^ GCE	1.431	1.093	0.674	0.653	1.309	0.021
^b^ GO/GCE	0.5843	0.309	0.676	0.651	1.890	0.025
^c^ NH_2_-Fe_3_O_4_ NPs-GO/GCE	2.396	1.929	0.678	0.657	1.242	0.021
^d^ ErGO/GCE	14.35	13.58	0.678	0.661	1.057	0.017
^e^ NH_2_-Fe_3_O_4_ NPs-ErGO/GCE	21.16	17.00	0.663	0.648	1.245	0.015

^a^ glassy carbon electrode; ^b^ graphene oxide modified glassy carbon electrode; ^c^ amine-functionalized Fe_3_O_4_ nanoparticles modified glassy carbon electrode; ^d^ electrochemically reduced graphene oxide modified glassy carbon electrode; ^e^ amine-functionalized Fe_3_O_4_ nanoparticles decorated electrochemically reduced graphene oxide modified glassy carbon electrode.

**Table 2:** Effect of different supporting electrolytes (0.1 M) on the current and potential response of 10 μM rutin at NH_2_-Fe_3_O_4_ NPs-ErGO/GCE. Accumulation time: 30s, other conditions are the same as in Figure 5.

Electrolyte	Peak Current (μA)	Peak Potential (V)
HAc-NaAc buffer (pH 4.0)	4.275	0.540
HAc-NH_4_Ac (pH 4.0)	4.645	0.564
(CH_2_)_6_N_4_-HCl (pH 5.0)	3.688	0.528
phosphate buffer (pH 6.0)	3.951	0.480
NH_3_-NH_4_Cl (pH 9.0)	1.264	0.432
HCl	6.200	0.648
HNO3	6.418	0.660
H2SO4	6.863	0.664
NaOH	0.645	0.328

**Table 3:** Influence of co-existing substances on the determination of 1.0 µM rutin (*n* = 3).

Coexisting Substance	Concentration/mM	Change of Peak Current/µA	RE/%	Coexisting Substance	Concentration/mM	Change of Peak Current/µA	RE/%
glucose	1	0.0546	1.28	uric acid	0.1	0.1761	4.13
citric acid	1	0.0674	1.58	Mn^2+^	1	−0.0942	−2.21
glutamate	1	0.1006	2.36	Zn^2+^	1	0.1530	3.59
arginine	1	0.1351	3.17	Ca^2+^	1	−0.1343	−3.15
tryptophan	0.1	0.1842	4.32	Mg^2+^	1	−0.0840	−1.97
tyrosine	0.1	0.1543	3.62	Pb^2+^	1	0.1100	2.58
glycine	1	0.0503	1.18	Cu^2+^	1	0.1778	4.17
lactose	1	0.0678	1.59	Al^3+^	1	−0.0972	−2.28
lactic acid	1	0.1006	2.36	K^+^	1	−0.0495	1.16
starch	1	−0.1215	−2.85	Na^+^	1	0.0622	1.46
sucrose	1	0.1177	2.76	PO_4_^3−^	1	0.0925	2.17
dopamine	1	0.2063	4.84	NO_3_^−^	1	0.0674	1.58
ascorbic acid	1	−0.1253	−2.94	Cl^−^	1	−0.0657	−1.54

**Table 4:** Comparisons among various electrochemical methods for rutin detection.

Electrochemical Sensors	Technique	Supporting Electrolyte	Linear Range/µM	Correlation Coefficient	Detection Limit/µM	References
^a^ GR–AuNPs/CSPE	^j^ SWV	HAc-NaAc buffer (pH 5.0)	0.1–15	0.9988	0.011	[12]
^b^ GR/CILE	^k^ DPV	PBS buffer (pH 2.5)	0.07–10; 10–100	0.997, 0.991	0.024	[13]
^c^ Cu–CS/MWCNT/GCE	DPV	B-R buffer (pH 3)	0.05–100	0.9994	0.01	[14]
^d^ BP–PEDOT:PSS/GCE	DPV	PBS buffer (pH 2.5)	0.02–15.0; 15.0–80.0	0.998, 0.996	0.007	[15]
^e^ GR-MnO_2_/CILE	DPV	PBS buffer (pH 2.5)	0.01–500.0	0.992	0.00273	[16]
^f^ AgNPs/PMB–GR/Au electrode	DPV	PBS buffer (pH 3.0)	0.01–0.1; 0.1–2.0; 2.0–10.0	0.9947, 0.9959, 0.9958	0.01	[17]
^g^ IL/CPE	SWV	B-R buffer (pH 3.29)	0.04–10	0.9984	0.01	[18]
^h^ MWCNTs–CHIT/ABPE	SDLSV	0.1 M HCl	0.02–10	0.9994	0.01	[19]
^i^ CTAB/ABPE	SDLSV	0.1 M HCl	0.006–0.2; 0.2–10	0.9985, 0.9992	0.004	[20]
NH_2_-Fe_3_O_4_ NPs-ErGO/GCE	SDLSV	0.2 M H_2_SO_4_	0.006–0.1; 0.1–8.0; 8.0–80		0.004	This work

^a^ graphene-gold nanoparticle carbon screen printed electrode; ^b^ graphene modified carbon ionic liquid electrode; ^c^ chemically cross-linked copper-complexed chitosan/multi-walled carbon nanotubes modified glassy carbon electrode; ^d^ black phosphorene- poly(3,4–ethylenedioxythiophene)–poly(styrenesulfonate) modified glassy carbon electrode; ^e^ graphene-MnO_2_ nanocomposite modified carbon ionic liquid electrode; ^f^ Ag nanoparticles/poly(methylene blue) functionalized graphene composite film modified Au electrode; ^g^ ionic liquid modified carbon paste electrode; ^h^ multi-walled carbon nanotubes–chitosan composite film modified acetylene black paste electrode; ^i^ cetyltrimethyl ammonium bromide film modified acetylene black paste electrode; ^j^ square-wave voltammetry; ^k^ differential pulse voltammetry.

**Table 5:** Rutin detection results from compound rutin tablets and urine samples (*n* = 4).

Sample	Labelled/µM	^d^ Detected by UV Method/µM	^d^ Detected by this method/µM	Added/µM	^d^ Total Found by this Method/µM	Recovery/%
^a^ Tablet 1	32.76	32.95 ± 0.94	31.48 ± 0.86	30.00	61.13 ± 2.14	98.8
^b^ Tablet 2	3.28	3.08 ± 0.11	3.37 ± 0.15	3.00	6.48 ± 0.20	103.7
Urine 1 ^c^	-	^e^ ND	ND	5.00	5.07 ± 0.18	101.4
Urine 2 ^c^	-	ND	ND	1.00	1.03 ± 0.03	103.0

^a^ Dilution factor: 1/100; ^b^ Dilution factor: 1/1000; ^c^ Dilution factor: 1/10; ^d^ Average ± confidence interval, the confidence level is 95%; ^e^ Not detected.
